# A qualitative exploration of the barriers and facilitators for engagement with a self-led biopsychosocial support tool, Pain Rehabilitation to Optimize Major Orthopaedic Trauma REcovery (PROMOTE), for patients with complex lower-limb orthopaedic trauma

**DOI:** 10.1302/2633-1462.76.BJO-2025-0395.R1

**Published:** 2026-06-02

**Authors:** Beth Fordham, Elizabeth Tutton, Rhys Painton, Jenny Gould, Juul Achten, Matthew L. Costa, David J. Keene

**Affiliations:** 1 Kadoorie Institute of Trauma, Emergency & Critical Care, Nuffield Department of Orthopaedics, Rheumatology and Musculoskeletal Science, University of Oxford, Oxford, UK; 2 Patient and Public Involvement and Engagement Group, Nuffield Department of Orthopaedics, Rheumatology and Musculoskeletal Sciences, University of Oxford, Oxford, UK; 3 Faculty of Health and Life Sciences, University of Exeter, Exeter, UK

**Keywords:** Orthopaedic trauma, Biopsychosocial, Qualitative, Intervention, Engagement, rehabilitation, Orthopaedic Trauma, lower-limb, randomized controlled trial, lower-limb trauma, chronic pain, lower-limb fracture, orthopaedic surgery, physiotherapists, healthcare professionals

## Abstract

**Aims:**

Pain Rehabilitation to Optimize Major Orthopaedic Trauma REcovery (PROMOTE) is a biopsychosocial intervention, delivered via a website format and supported by health professionals, to enhance the recovery of patients with complex lower-limb trauma. This qualitative study was embedded within a feasibility randomized controlled trial to explore experience and engagement.

**Methods:**

All participants randomized to PROMOTE, and staff delivering the intervention at four NHS major trauma centres, were invited to take part. An experienced qualitative researcher conducted online interviews with participants and focus groups with staff. Data were transcribed, managed in NVivo, and analyzed using framework analysis informed by the capability-opportunity-motivation model of behaviour.

**Results:**

Ten of 29 participants were interviewed, and 16 staff (four per site) participated in focus groups. Participant engagement ranged from accessing over 700 website pages to no use at all. Three key themes emerged to optimize engagement: 1) timing – delivery should occur after the immediate post-surgical period, when participants are “overwhelmed” but before they return to daily life and become too busy to prioritize their recovery; 2) format – content should be reduced, enhanced with audio and video, and be accessible offline; and 3) identity – PROMOTE should be introduced as a comprehensive recovery tool, not solely for pain or psychological wellbeing. Staff should receive enhanced biopsychosocial training to help them tailor the intervention to participants’ individual motivations.

**Conclusion:**

PROMOTE has the potential to address currently unmet needs for patients with complex lower-limb trauma; however, adjustments to timing, delivery format, identity, and staff training are required to maximize engagement and effectiveness in future trials.

Cite this article: *Bone Jt Open* 2026;7(6):724–732.

## Introduction

Annually in the UK, at least 20,000 people are affected by complex orthopaedic trauma,^1^ and 85% of these patients have fractures of a lower limb as part of their injuries.^2^ After a lower-limb fracture, patients are at high risk of developing problematic pain, defined as chronic pain associated with significant disability and/or distress.^3^ The majority (63%) of trauma patients still report moderately severe pain related to their injuries at one year post-injury,^4^ and the incidence of problematic pain may be as high as 85% in patients with multiple injuries.^5^ Managing problematic pain after complex trauma with medication (i.e. opioids) is not recommended and can lead to additional complications.^6^

The cognitive factors of low pain self-efficacy (confidence to engage in activities while in pain), low pain acceptance, high pain catastrophizing, and high pain-related fear avoidance are predictive of poor outcomes in this patient population,^7^ independent of the injury severity.^4^ Cognitive factors are amenable to modification with cognitive behavioural therapy (CBT) in chronic pain populations.^8^ The Pain Rehabilitation to Optimize Major Orthopaedic Trauma REcovery (PROMOTE) intervention was developed to deliver a low-intensity cognitive behaviourally informed intervention through a website platform to mitigate the risk of developing and/or impact of chronic pain. Concurrent to the PROMOTE research programme, researchers in North America have developed cognitive-behaviourally informed programmes for this patient population.^9,10^ These programmes were delivered face-to-face,^11^ via live video sessions,^9^ and via an app.^9^ All three programmes share similar core content and differ in how this information is communicated and delivered to the patient population. Therefore, it is key to understand why patients do or do not engage with the intervention, why this happens, and what we can do to improve.

The Behaviour Change Wheel is a guide to complex behaviour change intervention design based on a theory of human behaviour which was developed by synthesizing over 80 independent behaviour change theories.^12,13^ The underpinning theory is the capability-opportunity-motivation model of behaviour (COM-B), and this is a useful theory to understand barriers and facilitators for why people do or do not perform a behaviour, such as engaging with the PROMOTE website. Therefore, the COM-B model was used as a framework for analyzing the qualitative data regarding participant engagement with the PROMOTE website.

The aim of this study was to explore the barriers and facilitators for patients with complex lower-limb orthopaedic trauma, influencing engagement with the PROMOTE intervention.

## Methods

This qualitative study was embedded in the PROMOTE multicentre, parallel-group, feasibility randomized controlled trial.^14^

### Setting

Participants in the feasibility study were recruited from four NHS major trauma centres (MTCs; see Acknowledgements). Clinical teams identified potentially eligible patients after referral to the inpatient MTC service. Patients were screened and given study information within the first seven days after their orthopaedic surgery. Eligible patients who were keen to participate were approached for informed consent.

### Study participants

A convenience sampling technique was adopted to select participants from the quantitative feasibility study sample. All participants who were randomized to receive the PROMOTE intervention were approached to participate in this qualitative study. The initial approach was made face-to-face by the research team in the hospital. If they consented, participants were then telephoned by the researcher (BF) to arrange a time for a video interview. If BF could not contact participants by phone, she emailed participants to arrange a time for the interview. All healthcare professionals who were involved in delivering the PROMOTE intervention were invited to participate in the online staff focus groups.

The participant demographic characteristics from patients who participated in the PROMOTE intervention arm of the feasibility study, and those who participated in the qualitative study, are reported in Table I.

**Table I.** Participant characteristics.

Characteristic	PROMOTE intervention (n = 29)	Qualitative participants (n = 10)
Median age, yrs (IQR)	45 (32 to 62)	56 (45 to 66)
**Sex at birth, n (%)**		
Male	19 (66)	5 (50)
Female	10 (34)	5 (50)
**Index of Multiple Deprivation, n (%)**		
1 to 5	18 (62)	5 (50)
6 to 10	9 (31)	5 (50)
Unable to produce score	2 (7)	N/A
**Ethnicity, n (%)**		
White	19 (66)	9 (90)
Mixed or multiple	1 (3)	1 (10)
Asian or Asian British	4 (14)	0 (0)
Black African, Caribbean, or Black British	4 (14)	0 (0)
Other ethnic group	1 (3)	0 (0)

N/A, not applicable; PROMOTE, Pain Rehabilitation to Optimize Major Orthopaedic Trauma REcovery.

### Interviews

Our patient and public involvement and engagement (PPIE) group input into the research design suggested that patients would prefer one-to-one interviews and that, due to the nature of their injury, it would be more convenient to hold these via video call rather than meeting in person. Staff were happy to participate in focus groups as it facilitated conversations, and different staff members were more involved in different elements of the PROMOTE study, i.e. recruitment and delivery of the intervention.

The semistructured interview guides for the participant interviews and staff focus groups were designed by a senior researcher (ET) with prior experience of working with this patient group.^15-18^ The guides aimed to elicit responses regarding experience of participating in the PROMOTE trial with a particular focus on participants’ experience of engaging with the website.

The data were collected via video interviews (Microsoft Teams; Microsoft, USA) or via phone (digitally voice recorded) from patients at home and staff at work or at home, all via video interview. There were no other people present besides the participants and BF. The meeting was video recorded, and the audio data were downloaded, saved onto the University of Oxford’s secure drive, and transcribed manually. While transcriptions were available to participants, none chose to undertake member checking. The interviewer did not take field notes during the interviews or focus groups. There were no repeat interviews conducted.

### Research team and reflexivity

The participant and staff interviews/focus groups were all conducted by BF. The interviewer has a PhD in health psychology and over 15 years of experience in conducting qualitative interviews with participants and staff. At the time the interviews were conducted, she was employed as a health psychology research fellow at the Oxford Trauma and Emergency Care research group at the University of Oxford.

The interviewer (BF) did not have a relationship with the patient participants prior to the interviews. She did have a relationship with the staff, as she delivered the PROMOTE training to these sites as part of the PROMOTE feasibility study setup.

At the beginning of the interviews and focus groups, BF explained that she was not part of their medical care team and that she was an independent researcher who was interested in their perspectives on their care and the PROMOTE intervention. She explained that there were no right or wrong answers to these questions, that they would remain anonymous throughout, and that her role was to be an independent person feeding back the patient and staff experience.

The interviewer (BF) acknowledges holding a bias, as she was involved in the development of the PROMOTE intervention. BF engaged in reflexivity with the Trial Management Group (TMG), which included PPIE partners, in order to remain cognizant of her interest in the intervention and how it was received.

### Statistical analysis

**Fig. 1:**
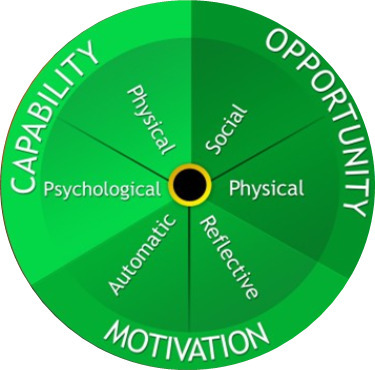
Capability-opportunity-motivation model of behaviour.^12^

The data were managed with NVIVO software and analyzed using a framework based upon the COM-B (Figure 1).^12,19^

Data were coded by BF and reflective discussions were held regularly with ET, a senior qualitative researcher who read all the transcripts. Interpretations of the data were explored and any differences discussed. Further reflexive discussions occurred with the research team and through presentation of the findings to the TMG which included PPIE partners (22 November 2024). These reflexive discussions were used to refine the analysis. Rigour was ensured through trustworthiness. Reflexive discussions, provision of an audit trail, and clarity regarding context and methods enable decision-making about transferability of the findings. The methods are reported in alignment with the COnsolidated criteria for REporting Qualitative studies (COREQ).^20^

## Results

**Fig. 2:**
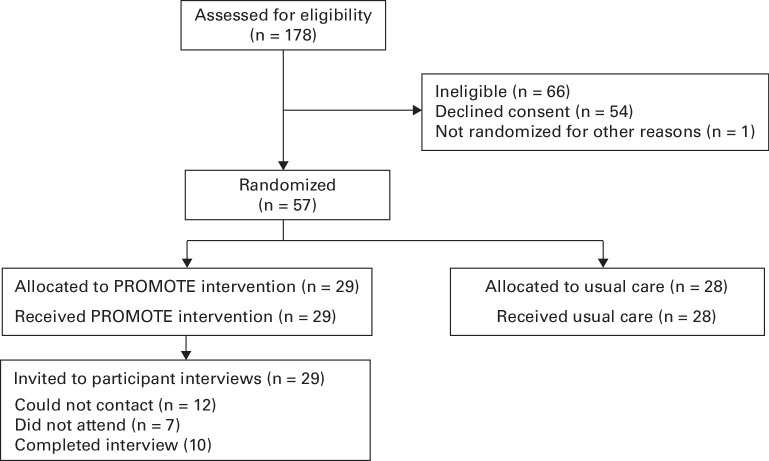
CONSORT flowchart for participants. PROMOTE, Pain Rehabilitation to Optimize Major Orthopaedic Trauma REcovery.

The PROMOTE feasibility trial recruited 57 participants between February and July 2024. Of the 29 participants who were randomized to participate in the intervention arm of the PROMOTE feasibility trial, ten (35%) consented and participated in the qualitative interviews. Participants were: five from Site 1, two from Site 2, two from Site 3, and one from Site 4. BF contacted and arranged an interview with an additional seven participants, but they did not attend the interview and could no longer be contacted via telephone or email. The remaining 12 participants from the intervention arm did not answer phone calls or emails. The CONSORT diagram is presented in Figure 2.

Staff from all four of the major trauma centres who participated in the trial also participated in the qualitative focus groups. Each site had four staff members (nurses and physiotherapists) attend the focus groups. One staff member, who was away when her site participated in the focus group, asked to be interviewed on a one-to-one basis on a separate date, which we did.

The participant interviews ranged from 30 minutes to one hour and 40 minutes in length, with most being approximately one hour in length. The staff focus groups ranged from 48 minutes to 58 minutes in length.

The participants in the interview study were older and more likely to be female than those in the PROMOTE arm of the feasibility study. Only one participant in the interview study was from a non-white ethnic group. A greater proportion of participants from higher socioeconomic groups (Index of Multiple Deprivation (IMD) scores)^21^ participated in the interview study than represented in the PROMOTE arm of the feasibility study. The interview sample were skewed in comparison to the feasibility trial sample but the sample was equally representative of females to males and lower to higher deprivation scores.

Participants and staff recognized that there is an unmet need for biopsychosocial support for this patient population. Participants were traumatized by their injury experience.

“I was very lucky to be alive and people were saying I was lucky and I kind of felt lucky to be alive but obviously it upset me.” (Site 3: Participant 01)

Participants were vulnerable and report being very distressed when they were inpatients.

“There were lots of other things that happened [as an inpatient] and it was very distressing to be honest.*”* (Site 1: Participant 26)

“That fear I was going to mess the bed and it is a fear, if you’re not used to being in that situation.” (Site 1: Participant 01)

All the participants reflected on the challenge of leaving inpatient care and having to manage daily life.

“So, what my conclusion is that while you’re in hospital you get the best treatment and when you’re in the big wide world you’re stuffed and you can quote me on that one.” (Site 1: Participant 25)

“I’ve done the easy bit, I fell asleep during the operation, I didn’t have to lift a knife, I’ve laid in bed, I’ve had my bum wiped for me, I’ve been fed and I’ve not had to do anything and so I’ve done all the easy bit. The next bit is I’ve got to get myself out of bed, I’ve got to try and get my legs which have done nothing for a month and get them to lift my fat arse and move round and it’s going to be incredibly difficult.” (Site 3: Participant 01)

Participants found a lot of the PROMOTE content very useful and helped them in their recovery.

“I find it – not a comfort but like …. reassurance.” (Site 3: Participant 07)

“PROMOTE has actually empowered me to be able to manage it and good because there is some information there to check in on, you know you can look at yourself.” (Site 1: Participant 16)

However, overall the participant engagement with PROMOTE website was low. Of the 29 participants allocated to receive PROMOTE, 22 (78.6%) accessed the website at least once. Participants accessed a median of 88 pages out of 275 pages (IQR 56 to 208).

The qualitative themes regarding participant engagement are represented within the COM-B model of behaviour framework. The COM-B model “is not a linear model in that components within the behaviour system interact with each other” and therefore, some barriers will interact with others.^12^

### Capability

Physical: Participants reported physical capability barriers to using the PROMOTE website during the early stage of their recovery. Some found it hard to hold their phone and read the content (delivery timing).

“If you need to read something there are so many muscles involved, so many things you need to do. Physically if you’ve broken your arm you can’t hold your phone and perhaps you need to read and you need glasses and if you don’t have glasses you can’t read.” (Site 1: Participant 11)

Staff suggested that the physical capability barrier of finding it hard to access the content via a website on their phone reduced the participant’s reflective motivation to engage (delivery format).

“I think these individuals are so invested in their own recovery and care, they don’t want to be in pain, they don’t want these ongoing issues, and they hear all the benefits and purposes of these sites and it’s absolutely ‘get me involved’. But then when you actually go through it, here’s your information sheet and that’s just pages long, and then here’s your website and it’s really how - on these tiny mobile phones. I think it’s so difficult to try and access.” (Site 4: Staff FG)

### Psychological

Psychological capacity refers to “the capacity to engage in the necessary thought processes – comprehension, reasoning etc”.^12^ Staff observed that participants often did not seem to have the ‘bandwidth’ or energy to engage with the website in the days following their surgery (delivery timing).

“It almost might be too soon, in like their recovery journey. They’re still in an acute hospital, in pain, maybe awaiting more surgery, unsure of events and everything else. Almost it was just another thing, and they just didn’t have the bandwidth [energy to think] I suppose to be like I need to go on this website and do this.” (Site 3: Staff FG)

“I think they just didn’t have the head space [energy to think] a lot of them to take on something else - when they’re in that emotional state.” (Site 4: Staff FG)

Participants explained that their pain medication made it hard for them to focus and comprehend the website in the early days following their surgery (delivery timing).

“Honestly, I was on so many drugs in the hospital and I had two generals [anaesthetics] and they kept on moving my bed at night, I was like off my head and it was loopy [chaotic].” (Site 1: Participant 16)

A clear emergent theme was that participants felt there was too much content for them to understand.

“I think one of the real barriers … has been just the amount of material was just too much. I think it lost a little bit of engagement.” (Site 2: Staff FG)

Other participants found the delivery format of a website to be a psychological barrier, as they could not use computers.

“I’m not very good with websites and all that sort of stuff and I’ve never been computer au fait… I wished I understood technology a bit better.” (Site 2: Participant 04)

Additionally, staff suggested the website delivery needed to be easier to use, especially for older adult users.

“I think that could be beneficial if it [the website] is a little easier to use for the patients and the older generation.” (Site 4: Staff FG)

One participant explained that because they had to work from home during their recovery, they were constantly on a computer screen. This was a psychological capability barrier to engaging with the content on a website (delivery format).

“I appreciate any time away from my laptop or anything that is digital now. So the PROMOTE thing for me was an extra thing… to deal with, to chat, to look at and to read on a screen.” (Site 1: Participant 11)

### Opportunity

Physical: A physical environmental barrier for participants, when they were inpatients, was a lack of reliable internet access on the trauma ward (delivery format).

“No, I couldn’t use the website in the hospital because the internet was bad… they do have WiFi but it just doesn’t work.” (Site 1: Participant 16)

Staff reflected that when patients were using the PROMOTE website, as an inpatient, they were often interrupted by clinical care (delivery timing).

“You know even if they were looking through a really interesting piece of material, they get interrupted because they’ve being taken for an X-ray or they’re having their observations taken. A lot of the control is not theirs.” (Site 2: Staff FG)

However, once participants returned home from the inpatient ward, they encountered another physical opportunity barrier, which was their lack of time (delivery timing).

“I just haven’t had time, I’ve been so busy working.” (Site 1: Participant 01)

Moreover, the time they had available was prioritized to activities which had greater motivational pull, such as finding a new job and juggling activities of daily living.

“Since I also lost my job and I couldn’t get the job I was supposed to start, because of this injury, I prioritize other things – aka [such as] find a job.” (Site 1: Participant 11)

“They were so enthusiastic when I met them but I think life gets in the way, maybe. As you say, it’s such a major injury that everything else will maybe be prioritized before they will go on to the app, but they are just my thoughts.” (Site 3: Staff FG)

Social: Social opportunity refers to an individual’s “cultural milieu that dictates the way we think about things”.^12^ Staff suggested that some of the participants did not see that the content of PROMOTE fitted with what they were expecting in their rehabilitation care (intervention identity).

“I think some of the patients actually said that to me, some of them said, well pain’s a problem but I don’t care about all this kind of mindfulness stuff.” (Site 2: Staff FG)

Some participants felt PROMOTE might be useful for other patients, but it did not fit with their perceived needs (intervention identity).

“I can’t really say it was irrelevant because it’s my own fault I haven’t used it to its full extent. So, I appreciate the fact that there’s an extra thing put in place [the PROMOTE intervention], which I think you guys should continue because you may have someone. I mean you need to think about your audience, right, think about the people you’re dealing with.” (Site 1: Participant 11)

Staff noticed that some participants were only motivated to engage with elements related to their physical recovery rather than their psychosocial recovery, possibly because psychosocial recovery is not connected to that individual’s cultural milieu.

“To be honest it was just a little bit disappointing in terms of the lack of engagement at that stage by the patients. Certainly, as has already been said, the physical side of their injuries seemed to outweigh any of the need to sort of look at the psychology or to look at the other factors.” (Site 3: Staff FG)

### Motivation

Reflective: By the nature of automatic motivation, which is unconscious to participants, we were only able to identify reflective or conscious motivational barriers to engagement. A clear theme was that the aim of PROMOTE to help patients with their pain was off-putting, because several participants did not view their pain as a major problem (intervention identity).

“I think the stress [of the PROMOTE intervention] was made how to manage pain but pain is the least of my problems.*”* (Site 1: Participant 11)

Staff suggested that the timing of introducing the possibility of problematic pain was demotivating for participants, as they were not yet experiencing problematic pain, so an intervention to help with pain was not relevant (intervention identity and delivery timing).

“We were introducing concepts to the patient about the study way too early, talking about the fact that they might have issues with pain, when they haven’t even got that far… when reality hits a bit then they’re going to be more receptive. They’re going to want to change things, rather than well, this might not happen. So, why am I going to engage with something that might not happen or I don’t want to happen.” (Site 2: Staff FG)

Staff highlighted that if they had more understanding of the contents of PROMOTE then they would have the skills to increase participant engagement with the website, tailoring their introduction to match the individual’s motivations more closely (intervention identity).

“I think our knowledge of what’s in this [PROMOTE intervention] and how it’s going to help this individual… then we can identify the stressor and ask the patient. Then we go, right we know the bits [sections] in here that are going to help with stress… I think that would help us know where we’re steering people and just give them that little bit more focus.” (Site 1: Staff FG)

Three core themes emerged across the COM-B framework analysis of participant engagement with the PROMOTE intervention: delivery format, delivery timing, and intervention identity. Table II presents these core themes and how these could be addressed in the next iteration of PROMOTE to increase participant engagement.

**Table II.** Core themes.

Theme	COM-B barrier	Quote	Solution
Delivery timing	Psychological capability	“I think they just didn’t have the head space.” “I was like off my head and it was loopy.”	Begin engagement with the website later in the inpatient stay: When patients have ‘head space’ Once the reality of recovery ‘hits’ Before they return home and become too busy
	Physical opportunity	“I just haven’t had time, I’ve been so busy working.”	
	Reflective motivation	“When reality hits a bit then they’re going to be more receptive.”	
Delivery format	Physical capability	“If you need to read something there are so many muscles involved, so many things you need to do.”	Change the format to: Rely less on written content Do not require participants to look at a screen Not need internet to access Make access as easy as possible
	Psychological capability	“I appreciate any time away from my laptop or anything that is digital now and so the PROMOTE thing for me was an extra thing…to deal with, to chat, to look at and to read on a screen.”	
	Physical opportunity	“I couldn’t use the website in the hospital because the internet was bad.”	
	Psychological opportunity	“I’m not very good with websites.” “I think that could be beneficial if it [the website] is a little easier to use for the patients.*”*	
Intervention identity	Social opportunity	“Pain’s a problem but I don’t care about all this kind of mindfulness stuff.”	Change the intervention identity to: Not be aligned with only pain Not be aligned with only psychological support
	Reflective motivation	“I think the stress [of the PROMOTE intervention] was made how to manage pain but pain is the least of my problems.*”*	

COM-B, capability-opportunity-motivation model of behaviour; PROMOTE, Pain Rehabilitation to Optimize Major Orthopaedic Trauma REcovery.

Participants offered some solutions for how to change the delivery format. One suggested delivering the content via audio, for example as a podcast, which participants could access without internet access and with their headphones in, which could reduce distraction.

“I think podcasts would be good, I mean but then I’m a 50-year-old woman, and I would like to listen to a podcast. I think they’ve all got their earphones and if it’s easily accessible I think that could be something that would work for them, I can see that that would be an easier option.” (Site 3: Staff)

Videos were easier for patients to access, and when they were delivered by a patient they were more engaging and relevant.

“I read a lot of the case ones where they showed the videos. The interesting thing was not just the video of the actual person who was talking about what had happened to him, it was after the video and this is what had happened since or what I expected during this time. Yes, which was really helpful as well. How they recover and the mechanisms they’ve got for coping with it and stuff. I found that useful.” (Site 3: Participant 07)

“I think the bitesize manageable kind of information giving is like a personal way, even if it’s watching a video of a person speaking. It’s definitely the way to go, I would say.” (Site 2: Staff)

One young participant (aged < 18 years) suggested delivering the information via a brief TikTok video.

“Obviously there’s like the new generation coming up and TikTok… I think if it’s in that sort of form, like short, snappy, quickfire.” (Site 2: Participant 05)

## Discussion

Some participants in the PROMOTE feasibility study engaged well with the website and accessed over 700 pages of content, whereas others did not access the website at all. This embedded qualitative study has identified a set of clear recommendations of how to increase participant engagement with the PROMOTE intervention. A challenge in delivering PROMOTE is to identify when the best time is to introduce a self-led biopsychosocial support tool to patients who have experienced a complex lower-limb trauma. If introduced too early, patients are overwhelmed and confused, especially when using pain medication; they are also unaware of the broader implications their injury has for their long-term recovery. However, once patients return home they are very busy with activities of daily life, which can make it less likely to become engaged with the intervention. It seems prudent to introduce the intervention to patients while they are still inpatients, but perhaps to allow experienced staff more autonomy in assessing when an individual will be more receptive and ‘ready’ to engage with PROMOTE.

The format for delivering biopsychosocial interventions in this clinical population has been examined in other research studies. Delivering an intervention face-to-face might engage more inpatients, but Vranceanu et al^22^ reported that patients would prefer receiving the support via a video delivery format. Delivering the intervention solely via an app did not engage this population.^11^ These findings are mirrored in our own findings, and the participants offer the suggestion that audio and video delivery could increase engagement with the content.

Finally, the identity of the intervention is important. Participants did not feel motivated to engage with an intervention which was focused on their pain, because their pain was well managed in early recovery. Nor were they drawn to an intervention which was to do with psychological wellbeing, as this did not seem socially acceptable within their cultural milieu. Therefore, the PROMOTE intervention needs to be marketed as a tool to support overall recovery, rather than having any specific connotations of pain or wellbeing.

The sociodemographic characteristics of participants in the qualitative study were not aligned fully with those of the participants in the feasibility study. The indicates potential selection bias, a limitation in this type of research where the experiences of those who do not participate cannot be incorporated. One factor that may have facilitated higher levels of engagement in this qualitative study is the time commitment required at a time later after injury, when social and financial impacts can be substantial. It is therefore critical that in the next iteration of PROMOTE we codesign the intervention and study process with patient and public representatives who are purposively selected from the sociodemographic groups that are currently under-represented in this research. We will ensure that there is support for appropriate payment, expenses, and recognition to reduce barriers to participation.

One very important finding was that staff were not consistently confident in how they should introduce the PROMOTE intervention to their patients. The intervention was designed with staff who were involved and invested in PROMOTE; however, we did not focus enough on how the intervention was introduced to staff and how we can motivate them and support them to deliver the intervention. This is a theme that we have identified as a major element to change in the next iteration of PROMOTE.

We recognize that the analysis was conducted by a single researcher in collaboration with other research team members but without systematic inter-rater reliability or coding verification. This stage was beyond the scope of our embedded qualitative study. Instead, our methodology draws upon validity and trustworthiness, relying on reflexivity as a tool for reliability. We hope that by using two highly experienced researchers, we have produced a robust set of findings that can help us in the next step of the research progression.

A strength of this study was that by using a behaviour change framework for analyzing the participant and staff data, we identified specific changes which can be made to increase engagement behaviour. For pragmatic reasons, we had one researcher who conducted and analyzed the interviews (BF) and we acknowledge that this could introduce bias. However, BF is a highly experienced qualitative researcher, and her findings were reviewed by a senior qualitative researcher who has extensive experience with this patient population (ET). BF also shared the analysis with the PROMOTE TMG to check for meaning and to challenge biases.

The content of PROMOTE is helpful for patient recovery after a complex lower-limb trauma. This paper has identified a set of recommendations for how the intervention delivery, format, and identity can be modified in the next iteration of PROMOTE to increase patient engagement.

**Take home message**

- Patients recovering from complex lower-limb orthopaedic trauma value biopsychosocial support, but engagement with digital interventions is limited by timing, format, and perceived relevance.

- Early post-injury recovery presents significant physical and psychological barriers to engaging with self-directed web-based interventions.

- Future iterations of PROMOTE should use flexible timing, simplified multimedia delivery, and frame the intervention as supporting overall recovery rather than pain management alone.

## Data Availability

The data that support the findings for this study are available to other researchers from the corresponding author upon reasonable request.
